# Toxoplasmosis of the spinal cord in an immunocompromised patient: case report and review of the literature

**Published:** 2013-12-31

**Authors:** Carolina Rodríguez, Ernesto Martínez, Guillermo Bolívar, Sandra Sánchez, Edwin Carrascal

**Affiliations:** 1 Universidad del Valle. Hospital Universitario del Valle. Department of Internal Medicine. Cali, Colombia. arpicaro@hotmail.com; 2 Universidad del Valle and Universidad Libre. Hospital Universitario del Valle, Department of Internal Medicine. Cali, Colombia. emarbui@gmail.com; 3 Universidad Libre, Department of Internal Medicine. Cali, Colombia. lunasa12@hotmail.com; 4 Universidad del Valle. Hospital Universitariodel Valle, Department of Pathology. Cali, Colombia

**Keywords:** Spinal cord toxoplasmosis, central nervous system, myelopathy in immunocompromised patient

## Abstract

We, herein, describe an HIV-positive patient with toxoplasmosis of the spinal cord. We also carried out a comprehensive literature review of this topic, with emphasis on the diagnostic tools and therapeutic approach.

## Abstract (es)

Se presenta el caso de un paciente seropositivo para VIH con diagnóstico de toxoplasmosis medular en conjunto con la revisión de la literatura de los pocos casos descritos hasta la fecha, con énfasis en las claves diagnósticas y la aproximación terapéutica.

## Introduction

Toxoplasmosis is the first cause of intracranial lesions associated to neurological deficit in HIV/AIDS¹. According to geographic location, the frequency varies between 3 and 50% of these patients. The risk factor most strongly related to this infection is CD4-positive lymphocyte count below 200/mm^3^, and especially below 50/mm^3^ ^3^ ^-^ ^6^. Extracerebral locations are described with less frequency, in less than 11% of the cases^2^ ^,^ ^6^, with myelitis due to *Toxoplasma* being an uncommon condition, with only 19 cases in medical literature, of which only seven have been confirmed in living patients^1^ ^,^ ^3^. Herein, we present the case of an HIV-infected patient diagnosed with myelitis due to *Toxoplasma* confirmed by biopsy, and review the published literature on this condition. 

Literature search was carried out in PubMed, Medline, LILACS, and SciELO databases by using the terms: *Toxoplasma*, toxoplasmosis, medullary, medular, spinal, myelitis, myelopathy. Descriptions in Spanish and English were considered of infection due to toxoplasmosis in spinal cord among adult patients. Two cases described in French were included.

### Case description:

The case was presented in the Internal Medicine Emergency Service at Hospital Universitario del Valle in Cali, Colombia. 

Clinical data was collected from the medical chart and signed informed consent was obtained from the patient for its publication. This was a 40 year-old, Latin American, heterosexual, male patient, with history of HIV infection diagnosed seven years ago. The patient was under antiretroviral treatment. His past medical history revealed an episode of cerebral toxoplasmosis five years ago, diagnosed through positive IgG for *Toxoplasma* and suggestive clinical presentation and scan imaging. This former episode was treated with standard dose of pyrimethamine and sulfadiazine with good clinical and images response, followed by intermittent prophylaxis with trimethoprim sulfamethoxazole. The patient presented to emergency room at our hospital with a two-year history of evolution of lumbar pain of moderate to severe intensity, associated to diminished strength in the lower limbs, more pronounced on the lower right limb, with compromise of the urinary sphincter during last months. The CD4 count was 60 cells/mm^3^ and the viral load was 55,110 copies/mL.

**Figure 1A:**
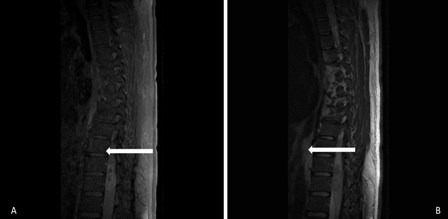
Thoracolumbar MRI. Phase contrasted with gadolinium, evidencing peripheral enhancement of the medullar lesion in segments T10 to T12, suggesting infection due to toxoplasma. 1B: Thoracolumbar MRI. T2 sequence, evidencing heterogeneous density lesion in medullar segments T10 to T12.

Physical exam revealed a patient in good nutritional condition, bedridden, with neurological deficit characterized by plegia in lower right limb, with greater compromise in distal roots of L3, L4, and L5 and paresis in the lower left limb. Further exam showed lack of bilateral Achilles and patellar reflex. Sensitivity was unaltered. The CSF extension exam resulted not suitable for cell count due to sample coagulation, with glucose of 6 mg/dL, proteins of 4,100 mg/dL, and LDH of 274 U/L. Magnetic resonance imaging (MRI) of thoracolumbar spine with gadolinium (Figs. 1A, 1B) showed an expansive lesion, with affectation of the distal medullary cone, isointense to spinal cord on T1, heterogeneous intensity, and areas of hyperintensity on T2. 

**Figure 2:**
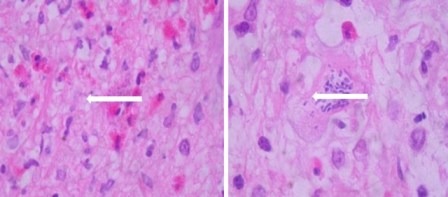
Microphotograph of medullary cone with extensive perivascular lymphocytic inflammatory infiltrate over a fibrillary stroma, with caseous necrosis, abundant eosinophils, and toxoplasma-type tachyzoite. H and E 100X

**Figure 3:**
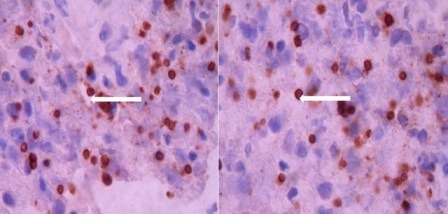
Microphotograph revealing numerous toxoplasma tachyzoites through immunoperoxidase reaction by using a specific monoclonal antibody against Toxoplasma gondii (Dako). 100X.

 The lesion extended from T10 to T12 and presented peripheral enhancement with contrast in relation to a probably infectious inflammatory process, suggesting toxoplasmosis as first possibility. Surgical exploration was conducted of the medullary cone, finding a thickened and hardened epiconus, with arachnoid and healthy skin, a tough avascular intra-axial fibrous lesion, from which samples were taken. The pathological study identified acute vasculitis with granulomatous component, extensive necrosis, and tachyzoites compatible with toxoplasmosis (Fig. 2). Special stains and cultures for acid fast bacilli and fungi were negative. The immunohistochemical study for *Toxoplasma* was positive (specific monoclonal antibody against *Toxoplasma gondii* - Dako) (Fig. 3). The PCR studies in CSF for herpes simplex virus types 1 and 2, Epstein-Barr virus, *Mycobacterium tuberculosis*, and cytomegalovirus were negative. Electromyography of the four limbs provided abnormal results, with electrophysiological evidence of motor polyneuropathy and distal axonal sensitivity in lower limbs. The B12 vitamin and plasma folate levels were normal. 

The patient received second-line treatment^7^ with trimethoprim sulfamethoxazole at a dose of 10 mg/k/d IV and clindamycin 1,200 mg IV every 6 h, according to the availability in the institution, with adequate tolerance. Clinical improvement was observed with partial recovery of his neurological deficit accomplishing deambulation with a walker aid at eight weeks of treatment, leaving him with a sequel of a right foot drop.

## Discussion:

Vacuolar myelopathy is a common condition with medullar compromise in HIV-positive patients, found in over 30% of autopsies prior the start of the era of antiretroviral therapy^1^. Other broadly described causes and possible differential diagnoses to bear in mind include HTLV I or II, herpes simplex 1 or 2, varicella zoster, cytomegalovirus, syphilis, and tuberculosis, among the infections; and lymphoma or nutritional deficiencies, among the non-infectious causes^6^ To date, only 18 cases have been described of myelitis due to *Toxoplasma* diagnosed histologically, via biopsy or autopsy, or through successful therapeutic trials within the context of a compatible clinical condition (Table 1). From the epidemiological point of view, these were patients almost all of male gender (90%), between the third and fourth decade of life. All the cases described have been associated to immunodeficiency, which only in three of these was not related to HIV. The symptoms described in most of the cases are lumbar pain, loss of motor function with compromise especially in lower limbs (70%), bladder dysfunction (55%), and sensitive alteration with specific medullar level (75%). One patient presented Brown-Sequard syndrome. In all cases in which a cerebrospinal fluid study was conducted, alterations were found, with increased protein levels being the most common finding, with values up to 2.2 g/dL. The IgG antibody *Toxoplasma* was positive in all but one of the patients evaluated. Magnetic resonance imaging (MRI) with gadolinium was the preferred imaging diagnostic method, with enhanced solitary intra-medullar lesions as the most frequent findings. The most frequently compromised segment was the thoracic (55%) and simultaneous cerebral and medullar involvement was established in half the cases described^1^ ^,^ ^3^. All the cases were immune suppressed patients, with the vast majority being HIV-positive with one case among these suspected medullary toxoplasmosis as a result of Immune Response Inflammatory Syndrome (IRIS)^2^ . With regards of treatment, similar courses of antibiotics to those considered standard for cerebral toxoplasmosis were given. According to current guidelines, variable results are obtained with pyrimethamine sulfadiazine as the first option, with early diagnosis being the best prognosis factor for complete recovery of these patients^4^. No special mention of using steroids associated to the antibiotic regime was found, although their use in two cases have been described with success. In our case, steroid treatment was administered during the first eight weeks of treatment, with good relative response in spite of the late diagnosis. More studies are needed to recommend this strategy in the future and determine the adequate manner for follow up and assessment of these patients.

**Table 1:**
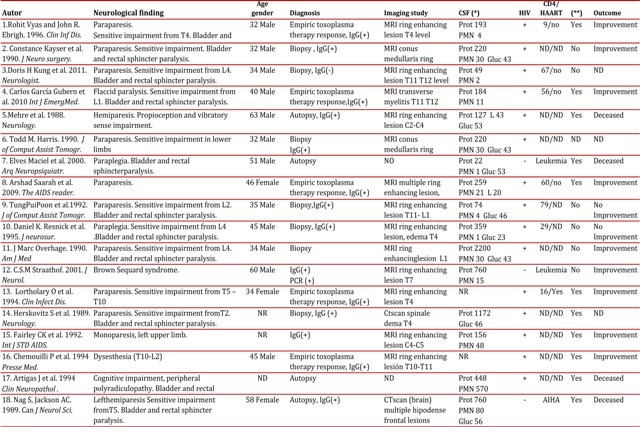

## Conclusion

In spite of the few cases described in the literature, myelitis due to *Toxoplasma gondii*could be a more common condition than thought. By being a treatable disease whose prognosis improves with early diagnosis, toxoplasmosis must be considered in the differential approach of all HIV-positive patients with suggestive clinical history, presence of medullar involvement during magnetic resonance study (especially if it is a solitary lesion), in combination with positive IgG antibody *Toxoplasma*. Timely treatment can result in the patient's significant improvement. 
